# Mitochondrial oligomers boost glycolysis in cancer stem cells to facilitate blebbishield-mediated transformation after apoptosis

**DOI:** 10.1038/cddiscovery.2016.3

**Published:** 2016-02-01

**Authors:** GG Jinesh, JR Molina, L Huang, NM Laing, GB Mills, M Bar-Eli, AM Kamat

**Affiliations:** 1 Department of Urology, The University of Texas MD Anderson Cancer Center, Houston, TX, USA; 2 Department of Systems Biology, The University of Texas MD Anderson Cancer Center, Houston, TX, USA; 3 Department of Cancer Biology, The University of Texas MD Anderson Cancer Center, Houston, TX, USA; 4 Astra Zeneca, Boston, MA, USA

## Abstract

Apoptosis culminates in secondary necrosis due to lack of ATP. Cancer stem cells form spheres after apoptosis by evoking the blebbishield emergency program. Hence, determining how blebbishields avoid secondary necrosis is crucial. Here we demonstrate that N-Myc and VEGFR2 control transformation from blebbishields, during which oligomers of K-Ras, p27, BAD, Bax, and Bak boost glycolysis to avoid secondary necrosis. Non-apoptotic cancer cells also utilize oligomers to boost glycolysis, which differentiates the glycolytic function of oligomers from their apoptotic action. Smac mimetic in combination with TNF-*α* or TRAIL but not in combination with FasL abrogates transformation from blebbishields by inducing secondary necrosis. Thus blebbishield-mediated transformation is dependent on glycolysis, and Smac mimetics represent potential candidates to abrogate the blebbishield emergency program.

## Introduction

Apoptosis culminates in secondary necrosis in most cell types.^1^ Cancer stem cells are capable of avoiding secondary necrosis and forming spheres (undergoing transformation) after apoptosis by evoking the blebbishield emergency program. The blebbishield emergency program exhibits two major phases: a blebbishield phase in which the apoptotic blebs fuse together to reconstruct the cells, and a sphere-formation phase in which blebbishields fuse to each other to form cancer stem cell spheres.^2^ The decision to undergo apoptosis or necrosis is determined by ATP production and thus by glycolysis;^3^ however, it is not yet understood how blebbishields avoid secondary necrosis.

Sphere formation is a hallmark of cellular transformation. Protein translation has a major role in transformation.^4^ Internal ribosome entry site (IRES) translation of proteins during cell death can rewrite the fate of cells: for example, c-Myc, c-IAP2, and XIAP are bona fide antiapoptotic IRES targets.^5^ Smac mimetics represent a novel class of small molecules that downregulate c-IAP2 and induce apoptosis with death ligands.^6,7^

Although apoptosis, glycolysis, IRES translational targets, and transformation are well studied, their role in blebbishield-mediated transformation is not yet understood. Using a Smac mimetic (AZ58) and TNF-*α*, TRAIL, and FasL, here we demonstrate that blebbishields increase glycolysis by forming mitochondrial oligomers of K-Ras, p27, BAD, Bax, and Bak to override secondary necrosis. Oligomers of these proteins in non-apoptotic cancer cells also boost glycolysis, which discriminates the glycolytic functions of oligomers from their apoptotic function. Transformation from blebbishields is mediated by N-Myc and VEGFR2. Caspase-3 targets the expression of VEGFR2 and N-Myc, inhibits glycolysis through Bax cleavage, and induces secondary necrosis in blebbishields to inhibit transformation. Thus glycolysis overrides secondary necrosis to aid blebbishield-mediated transformation and Smac mimetic plus TNF-*α* or AZ58 plus TRAIL represents a potential combination to target blebbishield-mediated cellular transformation.

## Results

### Downregulation of selected IRES translational targets and induction of necrotic PARP cleavage during apoptosis inhibits transformation from blebbishields

**Figure 1:**
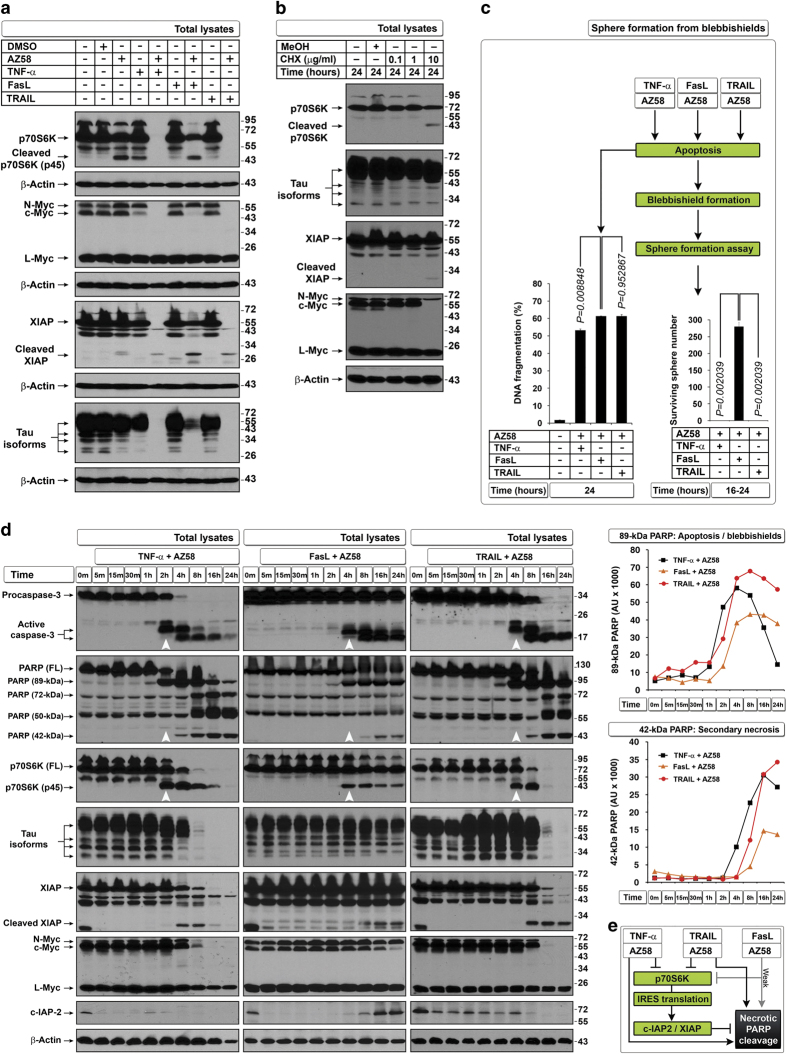
Defective downregulation of IRES targets and defective induction of secondary necrotic PARP cleavage leads to transformation from blebbishields. (**a**) TNF-*α* and TRAIL but not FasL in combination with AZ58 downregulate p70S6K and selected IRES translational targets at 24 h. (**b**) CHX 10 *μ*g/ml selectively targets c-Myc expression (also see Supplementary Figure S1A). (**c**) All three death ligands induce similar levels of DNA fragmentation in RT4v6 cells (left bar graph; *n*=3), but only blebbishields induced by FasL+AZ58 could transform (right bar graph; *n*=3). (**d**) Twenty-four hour pulse-chase analysis of timing of degradation of selected IRES translational targets in relation to caspase-3 activation (arrowheads), p70S6K and PARP cleavage (left blot panels), and quantification of apoptotic (89 kDa) and secondary necrotic PARP cleavage (42 kDa) by densitometry (right panels). (**e**) Schematic representation of lower efficiency of FasL than of TNF-*α* or TRAIL in combination with AZ58 in downregulating p70S6K and selected IRES targets in relation to secondary necrotic PARP cleavage.

We previously reported that bladder cancer cells (RT4 and RT4v6) are capable of blebbishield-mediated sphere formation (transformation) after apoptosis in a p70S6K expression–dependent manner^2^ and that Smac mimetic in combination with TNF-*α*, TRAIL, or FasL induces apoptosis through downregulation of c-IAP2 and XIAP (IRES translational targets).^6^ As IRES translation has pivotal roles in transformation^4^ and p70S6K regulates protein translation, we first examined the expression of p70S6K and a panel of known IRES translational targets that are influenced by apoptosis induced by Smac mimetic AZ58 in combination with TNF-*α*, TRAIL, or FasL in RT4v6 cells. We found that XIAP, c-Myc, N-Myc, and Tau (referred to as ‘selected IRES targets’ in this report) and p70S6K were drastically downregulated by the combination of AZ58 with TNF-*α* or TRAIL but not by the combination of AZ58 with FasL (Figure 1a). Both TNF-*α* and TRAIL plus AZ58 were efficient in downregulating c-Myc and N-Myc, while FasL plus AZ58 was able to downregulate only c-Myc (Figure 1a). Inhibiting protein translation using cycloheximide (CHX) at a dose of 10 *μ*g/ml also induced robust apoptosis (Supplementary Figure S1a) but without significant downregulation of selected IRES targets except c-Myc (Figure 1b). Despite the differences in degradation of selected IRES targets at 24 h after treatment, all three death ligands in combination with AZ58 induced pyknosis, blebbishield formation, and DNA fragmentation to a similar extent (Figure 1c and Supplementary Figure S1a). Interestingly, only blebbishields generated from FasL plus AZ58, not blebbishields generated from TNF-*α* or TRAIL plus AZ58, were capable of sphere formation (Figure 1c).

Because p70S6K controls protein translation by phosphorylation of ribosomal-S6 protein,^8^ we examined the levels of p70S6K (a caspase-3 substrate) in parallel to caspase-3 and its substrate PARP to confirm that the IRES targets were downregulated after the loss of p70S6K. Cleavage of p70S6K (resulting in p45 fragment) coincided with caspase-3 activation and 89-kDa PARP cleavage, whereas complete downregulation of full-length p70S6K coincided with downregulation of c-Myc, Tau, and XIAP (Figure 1d). Importantly, downregulation of full-length p70S6K, c-Myc, and Tau coincided with the generation of 50-kDa and 42-kDa necrotic PARP fragments, indicating the onset of secondary necrosis (Figure 1d).^9^ Intriguingly, FasL plus AZ58 did not generate necrotic PARP cleavage as efficiently as TNF-*α*- or TRAIL plus AZ58 (Figures 1d and e). Although FasL plus AZ58 induced caspase-3 cleavage as early as 4 h after treatment (similar to TRAIL plus AZ58 at 4 h), FasL plus AZ58 was able to downregulate only c-Myc at 24 h, raising the possibility that the cells managed to resume translation. To support this notion, c-IAP2 expression was restored after caspase-3 activation only in cells treated with FasL plus AZ58 but not in cells treated with TNF-*α* or TRAIL plus AZ58 (Figure 1d). Thus combinations of TNF-*α*, TRAIL, and FasL with AZ58 induce similar levels of apoptosis at 24 h but with different levels of downregulation of IRES translational targets and secondary necrotic 42-kDa PARP cleavage, resulting in transformation only from FasL plus AZ58-generated blebbishields.

### Downregulation of c-Myc is independent of caspase-3-mediated downregulation of other selected IRES translational targets

**Figure 2:**
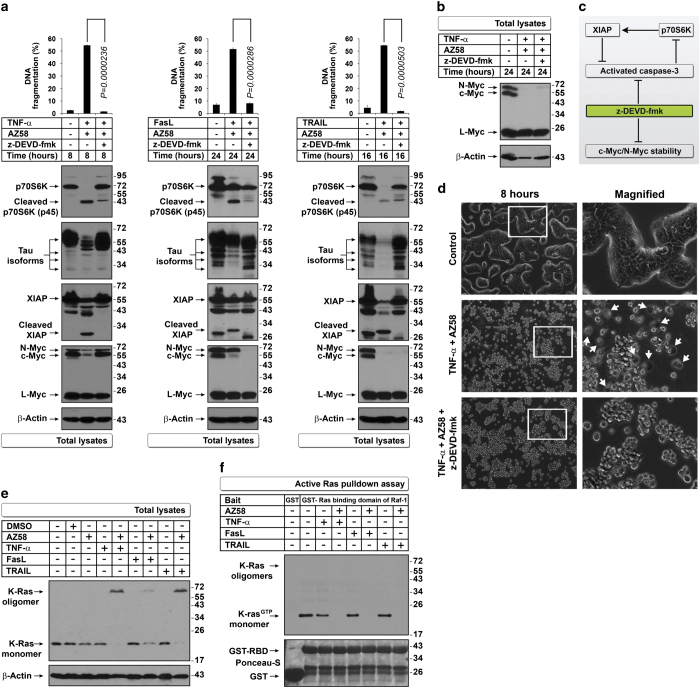
Downregulation of selected IRES targets is regulated by caspase-3 except for c-Myc, which is regulated by K-Ras inhibition. (**a**–**c**) Caspase-3 inhibition restores the expression of selected IRES targets but fails to protect c-Myc and N-Myc. Caspase-3 inhibitor z-DEVD-fmk restored Myc isoforms in TNF-*α*+AZ58-treated cells at (**a**) 8 h but failed to restore c-Myc and N-Myc at (**b**) 24 h. Bar graphs in panel (**a**) indicate that the DNA fragmentation was prevented by z-DEVD-fmk in all conditions (*n*=3). The simplified schematic diagram in panel (**c**) explains the action of z-DEVD-fmk on p70S6K and IRES targets. (**d**) Caspase-3 inhibition prevents secondary necrosis in blebbishields generated by TNF-*α*+AZ58 (arrows, secondary necrosis). (**e**) MW shift (oligomers) of K-Ras with FasL+AZ58 differs in quantity from MW shift with TNF-*α*+AZ58 or TRAIL+AZ58. (**f**) K-Ras activation assay. GST-Raf1-RBD binding to K-Ras was abolished by AZ58 combinations with TNF-*α*, FasL, and TRAIL.

Caspase-3 inhibition prevented downregulation of p70S6K, Tau, and XIAP and DNA fragmentation in all three combinations of AZ58 with death ligands but prevented downregulation of c-Myc only in the TNF-*α*-containing combination at 8 h but not at 24 h, indicating that c-Myc degradation could be caspase-3 independent (Figures 2a–c). Morphologically, TNF-*α* plus AZ58 induced secondary necrosis at 8 h, and this effect was prevented by z-DEVD-fmk (quantified below); despite pyknosis and formation of blebbishields were not prevented (Figure 2d and Supplementary Movie S1). These results, together with our finding that CHX selectively downregulated c-Myc to induce apoptosis, suggested that c-Myc downregulation is independent of caspase-3 activation and is a crucial event to induce apoptosis.

### Mitochondrial Bax p18 fragment but not oligomerization of K-Ras, p27, BAD, Bax, and Bak mediates mitochondrial outer membrane permeabilization (MOMP)

Ras signaling is known to stabilize c-Myc at the protein level,^10^ regulate p70S6K expression,^11^ and mediate cellular transformation through Myc.^12^ K-Ras is well known for its role in cellular transformation;^13,14^ hence, we examined K-Ras signaling. We identified a shift in molecular weight (MW shift) of K-Ras from 21 kDa to approximately 60–70 kDa under apoptotic conditions (Figure 2e). RT4v6 cells had constitutive K-Ras activation (21 kDa) that was abolished by combinations of TNF-*α*, FasL, and TRAIL with AZ58 at 24 h (both 21-kDa and 70-kDa shifted forms were not active; Figure 2f).

**Figure 3:**
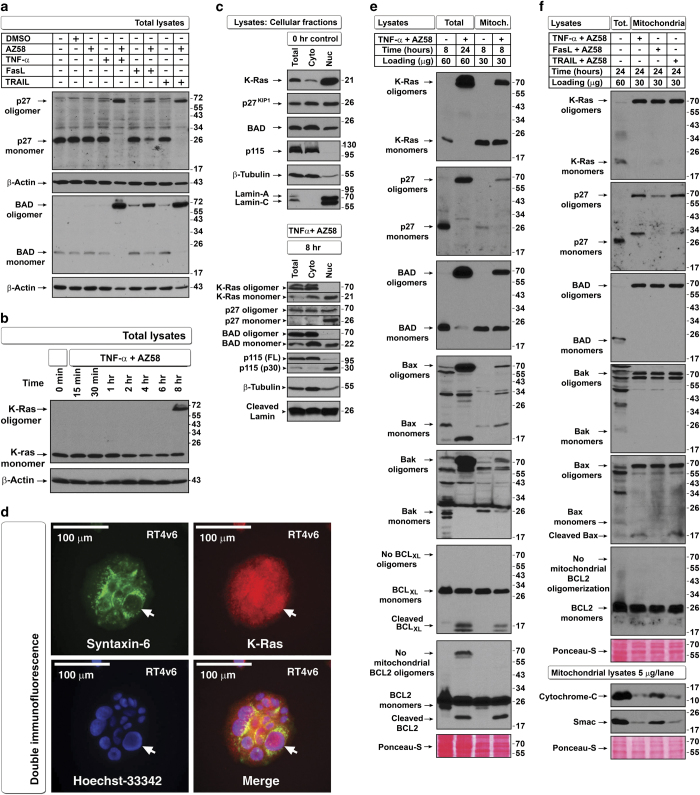
Cleaved Bax but not oligomers of K-Ras, p27, BAD, Bax, and Bak in apoptotic mitochondria correlates with inhibition of transformation from blebbishields. (**a**) TNF-*α* and TRAIL induce MW shift in p27 and BAD more efficiently than FasL+AZ58 does at 24 h time point examined by western blotting. (**b**) TNF-*α*+AZ58 induces MW shift in K-Ras as early as 8 h after treatment. (**c**) Nuclear and cytoplasmic fractionation of control cells (top panel) and TNF-*α*+AZ58-treated apoptotic cells at 8 h (bottom panel) to explore localization of K-Ras, p27, and BAD. No full-length lamin A/C was detected in treated apoptotic cells. (Note that cleaved 30-kDa fragment of p115 can enter the nucleus during apoptosis.^34^). (**d**) Double immunofluorescence confirmation of nuclear localization of K-Ras (arrows). Syntaxin-6 marks perinuclear Golgi membranes. (**e**) Localization of oligomers in mitochondrial fractions as early as 8 h after TNF-*α*+AZ58 treatment. Note that neither BCL_XL_ nor BCL2 from antiapoptotic members formed oligomers in mitochondria, although BCL2 oligomerized at non-mitochondrial sites. (**f**) Comparative mitochondrial oligomerization of K-Ras, p27, BAD, Bax, and Bak at 24 h and associated Smac and cytochrome-*C* release from mitochondria. FasL+AZ58 had reduced release of Smac and Cytochrome-*C*. First lane is total lysates except Smac and Cytochrome-*C*, which are control mitochondrial lysates.

The MW shift may be due to oligomerization as many apoptotic regulators are known to undergo oligomerization.^15^ Interestingly, p27 and BAD also exhibited similar MW shifts during apoptosis (Figure 3a). Upon treatment with TNF-*α* plus AZ58, the K-Ras MW shift occurred as early as 8 h (Figure 3b).

Under untreated conditions, K-Ras, p27, and BAD were localized in both cytoplasm and nucleus; however, under apoptotic conditions, the MW shift was restricted to the cytoplasm except for p27 (Figure 3c). We further confirmed that the nuclear fractions were not contaminated with Golgi membranes (where Ras can localize) by examining the endoplasmic reticulum–Golgi tethering protein p115 by western blotting and K-Ras nuclear localization by immunofluorescence (Figures 3c and d). As pro-apoptotic oligomers are known to localize to mitochondria, we examined K-Ras, BAD, and p27 localization along with known oligomerizing proteins Bax and Bak^16,17^ in isolated mitochondria from cells treated with TNF-*α* plus AZ58. At 8 and 24 h after treatment, K-Ras, p27, and BAD had undergone a MW shift to 70 kDa in mitochondria along with pro-apoptotic Bax and Bak oligomerization, demonstrating that K-Ras, p27, and BAD indeed undergo oligomerization similar to Bax and Bak (Figures 3e and f). We also detected a Bax p18 caspase cleavage fragment and loss of mitochondrial Smac and cytochrome-*C* in cells treated with TNF-*α* or TRAIL plus AZ58 but not FasL plus AZ58 (Figure 3f). Bax p18 is known to be more efficient in MOMP than intact Bax.^18^ We further detected K-Ras oligomers in mitochondria-depleted cytosol, demonstrating MOMP (Supplementary Figure S2a). Thus mitochondrial Bax p18 fragment but not oligomerization of K-Ras, p27, BAD, Bax, and Bak during apoptosis leads to MOMP.

### CHX enhances oligomerization of K-Ras, p27, BAD, Bax, and Bak and abolishes transformation from blebbishields by targeting N-Myc

**Figure 4:**
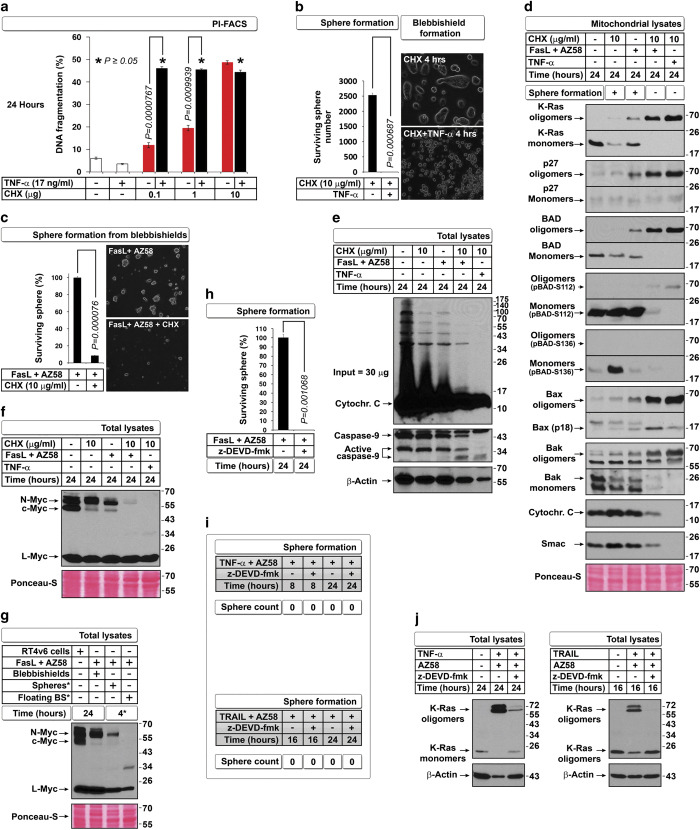
CHX and z-DEVD-fmk regulate oligomerization of K-Ras, p27, BAD, Bax, and Bak, and N-Myc expression but not oligomerization is required for transformation. (**a** and **b**) TNF-*α*+CHX induces robust apoptosis as analyzed by DNA fragmentation (**a**), but TNF-*α*+CHX-generated blebbishields do not form spheres (**b**). *P*≥0.05 in panel (**a**) indicates no significant differences in DNA fragmentation between three CHX dose combinations (*n*=3) (comparison of black bars only). Inset in panel (**b**) shows early induction of blebbishields by TNF-*α*+CHX. No treatments were carried out during transformation phase. (**c**) CHX inhibits transformation of FasL+AZ58-generated blebbishields. No treatments were carried out during sphere formation phase. (**d**–**f**) CHX not only enhances mitochondrial oligomerization of K-Ras, p27, BAD, Bax, Bak, and pBAD^112^ but also enhances Bax cleavage and release of Smac and cytochrome-*C* (**d**), resulting in differential caspase-9 cleavage (**e**) and loss of N-Myc and c-Myc (**f**). (**g**) N-myc is expressed in sphere-forming but not in non-sphere-forming (floating) blebbishields. *BS, blebbishields isolated after 24-h treatment; Spheres*, allowed transformation time 4 h. (**h** and **i**) Caspase inhibition abolishes transformation from FasL+AZ58-generated blebbishields (**h**) and fails to rescue sphere formation from TNF-*α*+AZ58- or TRAIL+AZ58-generated blebbishields (**i**). No treatments were carried out during sphere-formation phase. (**j**) Caspase inhibition blocks oligomerization of K-Ras.

CHX at a concentration of 10 *μ*g/ml alone (Supplementary Figure S1a) and CHX 10 *μ*g/ml in combination with TNF-*α* (as described previously^19^) induced equal apoptosis in RT4v6 cells (Figure 4a); however, only the blebbishields from CHX treatment alone were able to form spheres (Figure 4b). Microscopic analysis revealed that, compared with CHX alone, CHX plus TNF-*α* accelerated apoptosis at 4 h (Figure 4b) and induced secondary necrosis at 24 h (Supplementary Movie S2). In addition, CHX blocked sphere formation from FasL-plus-AZ58-generated blebbishields by 91.4% (Figure 4c).

We compared the mitochondrial oligomerization status of K-Ras, p27, BAD, Bax, and Bak between sphere-forming blebbishield-inducing conditions (CHX; FasL plus AZ58) and non-sphere-forming blebbishield-inducing conditions (CHX plus TNF-*α*; FasL plus AZ58 plus CHX) and found that mitochondrial oligomerization was significantly increased in non-sphere-forming blebbishields (Figure 4d). Correspondingly, we detected increased amounts of K-Ras oligomers in mitochondria-depleted cytoplasm, reflecting increased MOMP (Supplementary Figure S2b). As Ras signaling regulates the phosphorylation of BAD at Ser-112 to regulate glycolysis^20^ and glycolysis controls secondary necrosis,^3^ we examined the phosphorylation status of BAD. We found that BAD-Ser-112 phosphorylation was significantly reduced in non-sphere-forming blebbishield mitochondria compared with sphere-forming blebbishield mitochondria (Figure 4d). We also assessed MOMP by Bax p18 detection in mitochondria and loss of Smac and cytochrome-*C* from mitochondria. We found MOMP in non-sphere-forming blebbishields but not in sphere-forming blebbishields (Figure 4d). These data demonstrated that CHX in combination with TNF-*α* or FasL plus AZ58 blocks transformation from blebbishields by augmenting MOMP, which is accompanied by enhanced oligomerization of K-Ras, p27, BAD, Bax, and Bak. In this context, we had reported that mitochondrial depolarization leads to inhibition of transformation from blebbishields.^21^

Furthermore, CHX enhanced alternative processing of active caspase-9 (Figure 4e) and abolished N-Myc expression in non-sphere-forming blebbishields (Figure 4f). As loss of N-Myc correlated with loss of sphere formation (Figure 2b and Figures 2b and 4b, c, and f), we examined the requirement of N-Myc for sphere formation by fractionating sphere-forming and non-sphere-forming blebbishields from FasL-plus-AZ58-generated blebbishields after allowing 4 h for sphere formation and found that N-Myc was expressed only in sphere-forming blebbishields (Figure 4g). Together, these data demonstrated that CHX in combination with TNF-*α* or FasL plus AZ58 abolishes transformation by targeting N-Myc for degradation.

### Caspase-3 inhibition reveals that oligomerization of K-Ras is not required to block transformation from blebbishields

As loss of N-Myc leads to loss of transformation from blebbishields, we examined whether caspase-3 inhibition could block transformation from blebbishields, because z-DEVD-fmk abolishes N-Myc expression (Figures 2a and b). Caspase-3 inhibition abolished transformation from FasL-plus-AZ58-induced blebbishields (Figure 4h) and failed to rescue transformation from blebbishields induced by TNF-*α* plus AZ58 or TRAIL plus AZ58 (Figure 4i), which further confirmed the role of N-Myc in transformation from blebbishields. Next, we examined whether oligomerization of K-Ras has any role in inhibiting sphere formation from blebbishields. For this purpose, we chose combinations of TNF-*α* or TRAIL and AZ58 with or without z-DEVD-fmk (none of these conditions formed spheres). Caspase-3 inhibition blocked oligomerization of K-Ras (Figure 4j) but failed to rescue transformation (Figure 4i), demonstrating that oligomerization of K-Ras is not required to inhibit transformation from blebbishields.

### Oligomerization reflects energy demand and glycolytic status in blebbishields, but transformation is regulated by VEGFR2 and N-Myc

**Figure 5:**
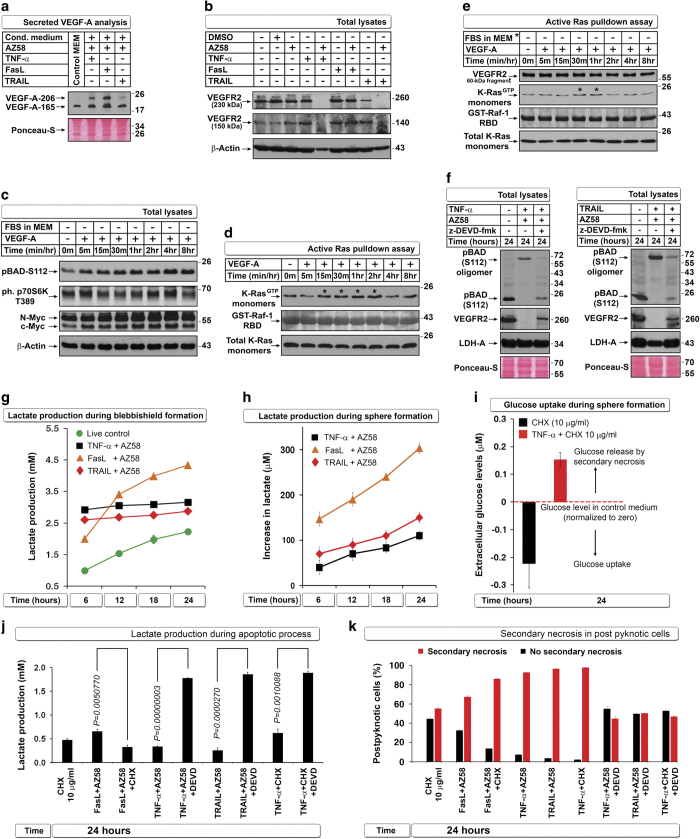
VEGF signaling regulates N-Myc stability and glycolysis to override secondary necrosis in blebbishields. (**a**) Death ligands in combination with AZ58 enhance secretion of VEGF-A isoforms detected by western blotting of conditioned media. (**b**) TNF-*α* or TRAIL but not FasL in combination with AZ58 targets VEGFR2 for degradation. (**c**) Recombinant VEGF-A induces pBAD^S112^ and phospho p70S6K and upregulates N-Myc and c-Myc in serum-free conditions (serum withdrawn 2 h before and during VEGF treatment). (**d** and **e**) K-Ras is constitutively activated in RT4v6 cells and upregulated (*) by VEGF in the presence of serum (**d**) or in serum-free conditions (**e**). A VEGFR2 fragment interacts with Raf-1 (**e**). Note: RT4v6 cells have autocrine VEGF signaling. (**f**) z-DEVD-fmk rescues loss of pBAD^S112^ and VEGFR2 induced by TNF-*α* or TRAIL in combination with AZ58. LDH-A: loading control. (**g** and **h**) FasL+AZ58 exhibits uninterrupted glycolysis during blebbishield formation (**g**) and sphere formation (**h**) as measured by lactate in conditioned media. (**i**) Glucose uptake measurement is not suitable for sphere-formation phase, as it is complicated by spillage of intracellular glucose by secondary necrosis. (**j** and **k**) CHX reduces lactate production and enhances secondary necrosis in combination with FasL+AZ58 or TNF-*α*; z-DEVD-fmk rescues lactate production and prevents secondary necrosis. Corresponding viability analysis is shown in Supplementary Figure S5.

Because K-Ras oligomerization is not required to inhibit transformation from blebbishields (Figures 4i and j) and because we previously reported higher sphere formation of VEGFR2^High^ cells than VEGFR2^Low^ cells,^2^ we investigated the VEGF signaling pathway. VEGF-A and VEGFR2 are required for transformation from blebbishields.^22^ TNF-*α*, FasL, and TRAIL plus AZ58 induced secretion of significant amounts of VEGF-A isoforms (Figure 5a); however, TNF-*α* plus AZ58 and TRAIL plus AZ58 but not FasL plus AZ58 abolished the expression of VEGFR2 at 24 h (Figure 5b). Recombinant VEGF-A (under serum-free conditions) induced increases in pBAD^112^, activated p70S6K, and N-Myc expression Figure 5c) and activated K-Ras in the presence or absence of serum (Figures 5d and e). Furthermore, z-DEVD-fmk rescued pBAD^112^ and VEGFR2 expression (Figure 5f), suggesting that VEGF signaling has a dominant role in blebbishield-mediated transformation.

Interestingly, K-Ras, p27, and BAD are all linked to glucose metabolism.^23–25^ Furthermore, p27 has a facultative role as a glucose sensor.^25^ Hence, we measured the lactate released from cells as a measure of glycolytic state. Formation of blebbishields was associated with higher lactate production compared with lactate production in non-apoptotic live cells (at 6 h); however, only blebbishields from the FasL plus AZ58 combination exhibited a steady increase in lactate production over time, indicating that only blebbishields from this condition continued to perform glycolysis (Figure 5g). Furthermore, glucose uptake was maximal in live control cells, whereas only blebbishields from the FasL plus AZ58 combination exhibited steady glucose uptake over time (Supplementary Figure S3a). Similarly, only blebbishields induced by FasL plus AZ58 exhibited a steady increase in lactate production during transformation (Figure 5h). Secondary necrosis in blebbishields leads to glucose spillage and increased the levels of glucose in medium above the levels present originally (Figure 5i). However, lactate measurements reflected the glycolytic state irrespective of secondary necrosis (Supplementary Figure S3b). Interestingly, caspase-3 inhibition rescued lactate production in blebbishields induced by combinations of TNF-*α* plus AZ58, TRAIL plus AZ58, and TNF-*α* plus CHX (Figure 5j).

**Figure 6:**
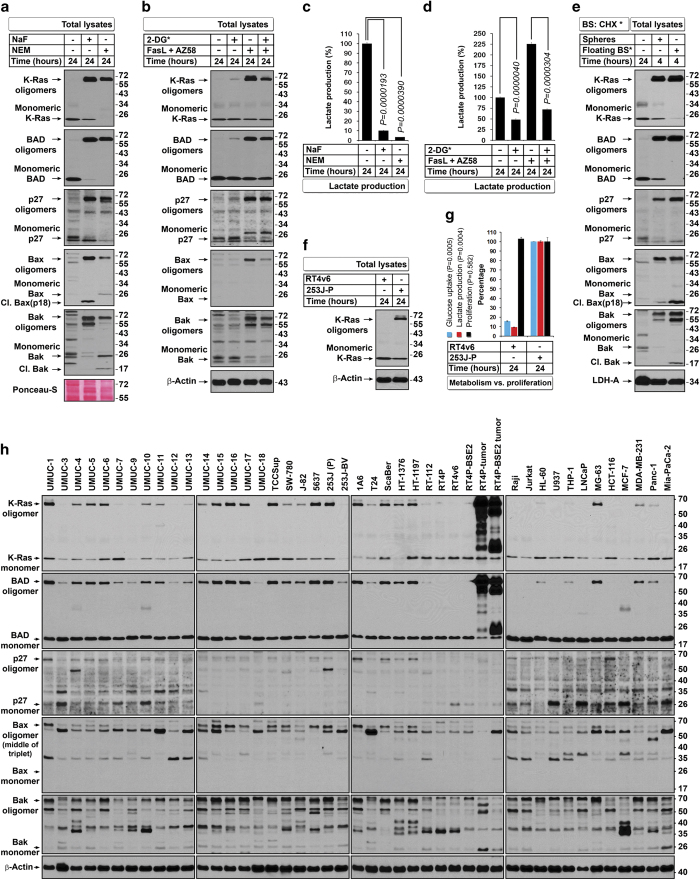
Oligomers of K-Ras, p27, BAD, Bax, and Bak boost glycolysis in blebbishields and non-apoptotic cells. (**a**) NaF and NEM enhance oligomerization of K-Ras, BAD, p27, Bax, and Bak. (**b**) 2-Deoxyglucose (2-DG*) induces oligomerization in non-apoptotic cells but interferes with oligomerization in FasL+AZ58-generated blebbishields. (**c** and **d**) NaF, NEM, and 2-DG* inhibit lactate production, reflecting inhibition of glycolysis. Controls considered 100%. Note that FasL+AZ58 increases glycolysis (apoptotic energy demand) (**c**) as well as oligomerization (**b**) (*n*=3). (**e**) Oligomers are expressed in both sphere-forming and non-sphere-forming blebbishields (floating BS*, floating blebbishields); monomers are expressed only in sphere-forming blebbishields. *BS: CHX, blebbishields generated by treatment with CHX 10 *μ*g/ml for 24 h. (**f** and **g**) Oligomers represent highly glycolytic cancer cells under non-apoptotic conditions. 253 J-P cells constitutively express K-Ras oligomers (**f**) and have increased glucose uptake and lactate production although proliferation differences were insignificant (**g**). 253 J-P measurements were considered 100% (*n*=3). (**h**) Oligomers are widely expressed in cancer cells under non-apoptotic conditions and *in vivo* (tumor samples: BSE2, tumors from blebbishield-derived cells). Bax reference band (arrow) is the middle of a triplet of oligomers of UMUC-15 that also correlates with K-Ras and BAD patterns across cell lines (also refer to Bax oligomers in panel (**a**)).

As glycolysis controls ATP production and ATP determines secondary necrosis, we examined the level of secondary necrosis in isolated post-pyknotic cells and found that low lactate production in blebbishields correlated with higher secondary necrosis and release of cytochrome-*C* into medium, whereas z-DEVD-fmk reversed both secondary necrosis and cytochrome-*C* release into media (Figure 5k compared with Figure 5j and Supplementary Figure S3c). The blebbishields that lacked secondary necrosis were indeed viable (Supplementary Figure S4). These data suggested that oligomerization of K-Ras, p27, and BAD reflects the glycolytic energy demand to meet the apoptotic events and that caspase-3 inhibition minimizes the apoptotic energy demand and thereby minimizes the need to form oligomers of K-Ras, p27, and BAD. We blocked ATP production in RT4v6 cells by previously reported agents such as NaF and *N*-ethylmaleimide (NEM)^26^ to examine whether oligomerization is related to ATP availability. We found that both agents induced robust oligomerization of K-Ras, p27, BAD, Bax, and Bak with associated blockade in lactate production, demonstrating that ATP paucity owing to glycolytic shut-down is the cause of oligomerization (Figures 6a and c). To further examine whether interfering with glycolysis using a specific inhibitor of glycolysis could form oligomers, we used 2-deoxyglucose (2-DG; introduced in MEM), 1 : 1 with normal glucose (MEM component). We found that 2-DG could generate oligomerization of K-Ras, p27, and BAD in non-apoptotic cells (Figure 6b). However, under FasL-plus-AZ58-induced apoptotic conditions, 2-DG reduced oligomerization as well as lactate production (Figures 6b and d), demonstrating the existence of a feed-forward loop during apoptotic oligomerization.

Furthermore, sphere-forming blebbishields expressed oligomers of K-Ras, p27, BAD, Bax, and Bak, demonstrating that these oligomers are not detrimental to sphere-forming blebbishields (Figure 6e). However, the non-sphere-forming blebbishields expressed cleaved Bax p18 and Bak p18 fragments, suggesting the importance of MOMP in blebbishield death (Figure 6e).

K-Ras is known to augment glycolysis in transformed cells,^24^ and some cell types are highly glycolytic under non-apoptotic conditions.^27^ We found that 253-J cells (parental cells: 253 J-P) exhibited constitutive oligomerization of K-Ras and had 10-fold higher glycolytic rate and glucose uptake than RT4v6 cells at equal cell density (RT4v6 cells do not have constitutive K-Ras oligomerization) (Figures 6f and g). We also screened 43 cancer cell lines (excluding tumor samples) from various cancer types and found that 32 of these cell lines (74%) exhibited constitutive K-Ras, p27, BAD, and Bax oligomerization (Figure 6h). Together, these data demonstrated that oligomerization boosts glycolysis in apoptotic and non-apoptotic cells.

## Discussion

**Figure 7:**
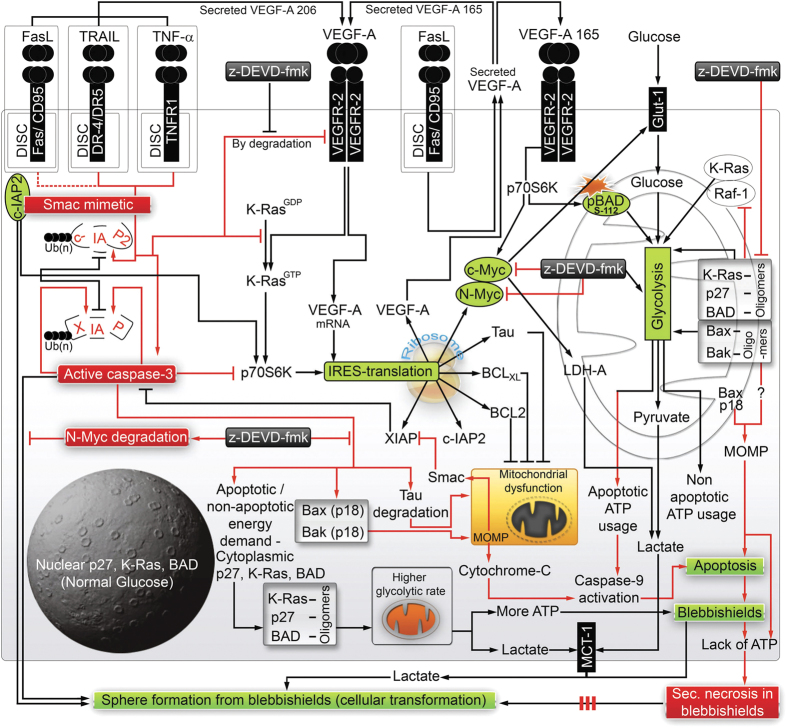
Schematic diagram showing intricate network of cytokines and glycolysis regulating life (transformation) and death decisions of blebbishields. FasL+AZ58 is a weak signal leading to weaker degradation of c-IAP2 (red dotted line). The solid red lines show the events that lead to demise of blebbishields by MOMP, caspase-3 and caspase-9 activation, glycolytic shut-down, ATP depletion, and secondary necrosis. Black arrows indicate survival signals. Although z-DEVD-fmk rescues glycolysis, VEGFR2 expression, and many IRES targets, it does not rescue transformation from blebbishields as it targets c-Myc and N-Myc expression. Red boxes, key destructive signals. Green boxes, key survival signals.

Our study evaluated the intricate network of apoptosis, glycolysis, IRES translational targets, and cellular transformation in the context of blebbishields as summarized in Figure 7. We found that FasL differs from TNF-*α* and TRAIL in that FasL supports transformation from blebbishields. In support of this notion, acquisition of FasL and Fas expression in cancers might be an important cause of recurrence after therapy, possibly through blebbishield emergency program.^28^

In the current study, we identified the important roles of oligomers in boosting glycolysis to override secondary necrosis in blebbishields and in non-apoptotic cancer cells. K-Ras signaling is known to induce pBAD^112^ and glycolysis^20^ and mediate cellular transformation by metabolic switch in collaboration with p27, BAD, N-Myc, VEGFR2, and Raf-1 (Supplementary Table S1); all of these molecules have a central role in blebbishield-mediated transformation. Hence, we conclude that blebbishield emergency program is an essential intermediate step in K-Ras-mediated cellular transformation.

Oligomerization of K-Ras, p27, BAD, Bax, and Bak in non-apoptotic cells demonstrates that the oligomers observed in this study are for glycolytic function than for mediating MOMP. MOMP induced by Bax p18 fragment is another crucial aspect in blocking the blebbishield emergency program.

Overall, our study uncovered the importance of glycolysis and its regulation by mitochondrial oligomers of K-Ras, p27, BAD, Bax, and Bak in preventing secondary necrosis to facilitate N-Myc- and VEGFR2-mediated transformation from blebbishields and identified AZ58 plus TNF-*α* and AZ58 plus TRAIL as potential therapeutic combinations to target blebbishield-mediated cellular transformation.

## Materials and Methods

### Cells and maintenance

Human RT4v6 bladder cancer cells and non-bladder cancer cell lines were described previously.^2^ Various bladder cancer cell lines were kindly provided by Dr. David J McConkey (Department of Urology, The University of Texas MD Anderson Cancer Center, Houston, TX, USA). All cells were maintained in MEM/RPMI with 10% fetal bovine serum, L-glutamine, pyruvate, non-essential amino acids, vitamins, penicillin, and streptomycin.

### Antibodies

Antibodies to p27 (2552), phospho S-235/236 S6 ribosomal protein (2211), XIAP (2045), c-Myc (5605), p70S6K (2708), Syntaxin-6 (2869), *β*-Tubulin (2146), pBAD-S112 (9296), pBAD-S136 (9295), ph-p70S6K T389 (9206), and LDH-A (3582) were purchased from Cell Signaling Technology (Beverly, MA, USA). Antibodies to Cytochrome-*C* (S2050) and c-IAP-2 (51-9000062) were purchased from BD Biosciences (San Jose, CA, USA). Antibodies to Tau (Sc-21796), BCL2 (Sc-509), BCL_XL_ (Sc-8392), K-ras (clone F234) (Sc-30), PARP-1 (Sc-7150), p115 (Sc-48363), Lamin A/C (Sc-7292), Bax (Sc-526), Bak (Sc-7873), VEGF-A (Sc-7269), VEGFR2 (Sc-504), Caspase-9 (Sc-7885), and Caspase-3 (Sc-7148) were purchased from Santa Cruz Biotechnology (Santa Cruz, CA, USA). BAD antibody (B31420) was purchased from Transduction Laboratories (Lexington, KY, USA). Antibody to Smac (AF7891) was purchased from R&D Systems (Minneapolis, MN, USA).

### Reagents

Caspase-3 inhibitor z-DEVD-fmk (FMK004; used at 20 *μ*M throughout) and recombinant cytokines TNF-*α* (210-TA) (used at 17 ng/ml throughout), FasL (126FL/CF; used at 17 ng/ml throughout), TRAIL (375-TL; used at 17 ng/ml throughout), and VEGF-A (293-VE-10; used at 19 ng/ml throughout) were purchased from R&D Systems. 2-Deoxyglucose (D8375), NaF (201154), and NEM (E3876) were purchased from Sigma (St Louis, MO, USA). Live/Dead Assay Kit (L3224) was purchased from Invitrogen (Carlsbad, CA, USA). CHX (239763) was purchased from Calbiochem (San Diego, CA, USA). AZ58 is a proprietary Smac mimetic^29,30^ provided by Astra Zeneca (Boston, MA, USA).

### Western blotting and densitometry

Cells were lysed using whole-cell lysis buffer (50 mM Tris-HCL, pH 7.4; 150 mM NaCl; 5 mM EDTA; 25 mM NaF; 1% Triton-X 100; 1% NP-40; 0.1 mM Na_3_VO_4_; 12.5 mM *β*-glycerophosphate; 1 mM PMSF, and complete protease inhibitor cocktail (Roche)) by incubation on ice for 30–40 min with intermittent vortexing every 10 min. The lysates were clarified at 13 000 r.p.m. for 10 min, and the supernatants were quantified and subjected to SDS-PAGE and western blotting on nitrocellulose membranes. The 5x SDS-PAGE sample buffer used was as follows: 375 mM Tris, pH 6.8; 0.01% bromophenol blue; 10% glycerol; 2% SDS; and 12.5% *β*-mercaptoethanol. Blot images were quantified using the ImageJ software v1.45 s (NIH, Bethesda, MD, USA) where appropriate.

### Blebbishield-mediated transformation assay

Cell were plated at a density of 200 000 cells/ml (10 ml per 100-mm plate or 15 ml per T-75 flask) and treated 24 h later as indicated in the figures. Twenty-four hours after treatment (or at the times indicated in figures), the floating pyknotic cells were collected, pelleted at 1200 r.p.m. for 3 min at room temperature, and re-plated in triplicate with complete MEM for a further 16–24 h. The floating cells were then washed off, and the attached spheres were counted.

### Isolation of mitochondria and mitochondria-depleted cytoplasm

Mitochondria and cytosolic fractions were isolated by differential centrifugation as described previously.^31^ For depleting mitochondria from cytosol, the cytosolic fraction was clarified at 13 000 r.p.m. for 10 min. Mitochondria were lysed with whole-cell lysis buffer (as described in the Western blotting section).

### Preparation of nuclear and cytoplasmic extracts

RT4v6 cells were plated in 10-cm plates at a density of 50 000 cells/ml and treated with or without TNF-*α* plus AZ58 for 8 h. The cells were then subjected to nuclear and cytoplasmic extract fractionation using the NE-PER Nuclear and Cytoplasmic Extraction Kit (Pierce: 78833; Rockford, IL, USA) per the manufacturer’s instructions.

### Quantification of DNA fragmentation

RT4v6 cells (50 000/ml, 4 ml/well) were plated in six-well plates and treated 24 h later with various cytokines and chemicals as indicated in the figures for the indicated durations. Both floating cells and trypsinized adherent cells were subjected to PI-FACS as described previously.^6^

### Quantification of secondary necrosis

RT4v6 cells were treated with or without various cell death-inducing treatments as indicated in the figures for 24 h, and the floating pyknotic cell populations were isolated (trypsin was avoided throughout the protocol except for excluding auto-fluorescence and live control cells). Cells were pelleted down at 3500 r.p.m. for 5 min, and cells were resuspended and incubated for 20 min in 100 nM calcein-AM and 8 *μ*M ethidium homodimer-1 containing PBS at room temperature (Live/Dead Cytotoxicity Kit, Invitrogen) before analysis by flow cytometry. The FACS files were merged using the Flowjo software (Ashland, OR, USA).

Secondary necrotic PARP cleavages after apoptotic PARP cleavage (50 and 42 kDa after 89 kDa) were quantified using western blotting and densitometry as described in the Western blotting section.

### Glycolysis and oligomerization analysis

For glucose metabolism studies, 200 000 cells were plated and treated as indicated in the figures, and blebbishields were isolated as described above. The conditioned media at the indicated time points were collected separately for metabolite analysis as described below. Blebbishields were counted and plated further in complete MEM at a density of 100 000 blebbishields/ml; conditioned media were collected and clarified at 3500 r.p.m.; and the supernatants were frozen until analysis. The conditioned media were examined for the levels of glucose and lactic acid using YSI 2900 Biochemistry Analyzer (YSI Life Sciences, Yellow Springs, OH, USA) per the manufacturer’s instructions. Increase in lactate production was calculated by subtracting the lactate present in the control medium (without exposure to cells; the lactate is from FBS) from the lactate present in the experimental media. Glucose uptake was calculated by subtracting the glucose levels of the experimental media from the glucose level of the control medium.

For oligomerization studies, the cells were induced for apoptosis using FasL plus AZ58 with or without 5 mM 2-DG (in MEM that had equal molar normal glucose) or 20 mM NaF and 1 mM NEM before immunoblotting. For experiments comparing RT4v6 with 253 J-P cells for lactate production, 200 000 cells/ml were plated, and 24 h later, conditioned media were subjected to glucose and lactate measurement, and the cells were counted to examine the differences in proliferation.

### K-Ras activation assays

Active ras pull-down assay was performed as previously described with minor modifications. GST expression plasmid Raf1-RBD (Ras binding domain)-aa1-149 (Dr. Channing Der; Addgene-13338; described in Brtva *et al*.^32^) and control GST (a gift from Dr. Santosh Chauhan, The University of New Mexico, USA) were induced in DH5*α* with 1 mM IPTG for 4 h at 37 °C, and the GST fusion proteins were purified as described previously.^33^ Two hundred micrograms of cell lysate in 200 *μ*l of whole-cell lysis buffer per condition was diluted with 200 *μ*l of magnesium-containing lysis buffer (MLB: 25 mM HEPES, pH 7.5; 150 mM NaCl; 1% (w/v) NP-40; 0.25% (w/v) sodium deoxycholate; 10% glycerol; 20 mM MgCl_2_; 1 mM EDTA; freshly added protease inhibitor cocktail (Roche); and 1 mM PMSF) and subjected to pull-down using freshly purified GST/GST-RBD for 1 h at 4 °C. The bound proteins were washed six times using MLB and subjected to SDS-PAGE and western blotting for the target proteins as indicated in the figures.

### Double immunofluorescence

Cells were fixed at room temperature for 15 min (4% paraformaldehyde in PBS), washed, blocked (blocking buffer: 1% BSA and 0.3% Triton-X100 in PBS) for 1 h, incubated with primary antibodies for 16 h at 4 °C (K-Ras 1 : 50 and Syntaxin-6 antibodies 1 : 100 in blocking buffer), washed, incubated with secondary antibodies for 1 h at room temperature (Alexa-555 anti-mouse and Alexa-488 anti-rabbit; Invitrogen), washed, and incubated with 10 nM Hoechst-33342 (Sigma) for 20 min before imaging.

### Two-dimensional imaging and time-lapse microscopy

All imaging studies were performed with an Olympus IX81 microscope equipped with heating stage (37 °C) and humidified CO_2_ chamber (5%). Image processing was carried out as described previously.^2^

### Statistical analyses

Statistical analyses were performed using Microsoft Excel 2010. The statistical significance was determined based on Student’s *t* test with two-tailed distribution and two-sample unequal variance, and *P*-values <0.05 were considered significant. The error bars represent S.Es., except the error bars for glucose and lactate measurements, which represent S.Ds.

## Abbreviations

c-IAP2: cellular-inhibitor of apoptotic protein-2
IRES: internal ribosome entry site
MOMP: mitochondrial outer membrane permeabilization
p70S6K: 70 kDa ribosomal S6 kinase
XIAP: X-linked inhibitor of apoptosis
VEGF-A: vascular endothelial growth factor-A
TNF-*α*: tumor necrosis factor-*α*
TRAIL: tumor necrosis factor related apoptosis inducing ligand
FasL: Fas ligand
PARP: poly ADP ribose polymerase
GST: glutathione S-transferase
LDH-A: lactate dehydrogenase-A.
