# Supplementary material for: Mitochondrial oligomers boost glycolysis in cancer stem cells to facilitate blebbishield-mediated transformation after apoptosis
Source: Cell Death Discov. 2016 Feb 1;2:16003–. doi: 10.1038/cddiscovery.2016.3 (PMC4979437; doi:10.1038/cddiscovery.2016.3)
Supplement: Supplementary Information [file cddiscovery20163-s1.doc]

**Supplemental information**

**Supplemental figures and legends**

**
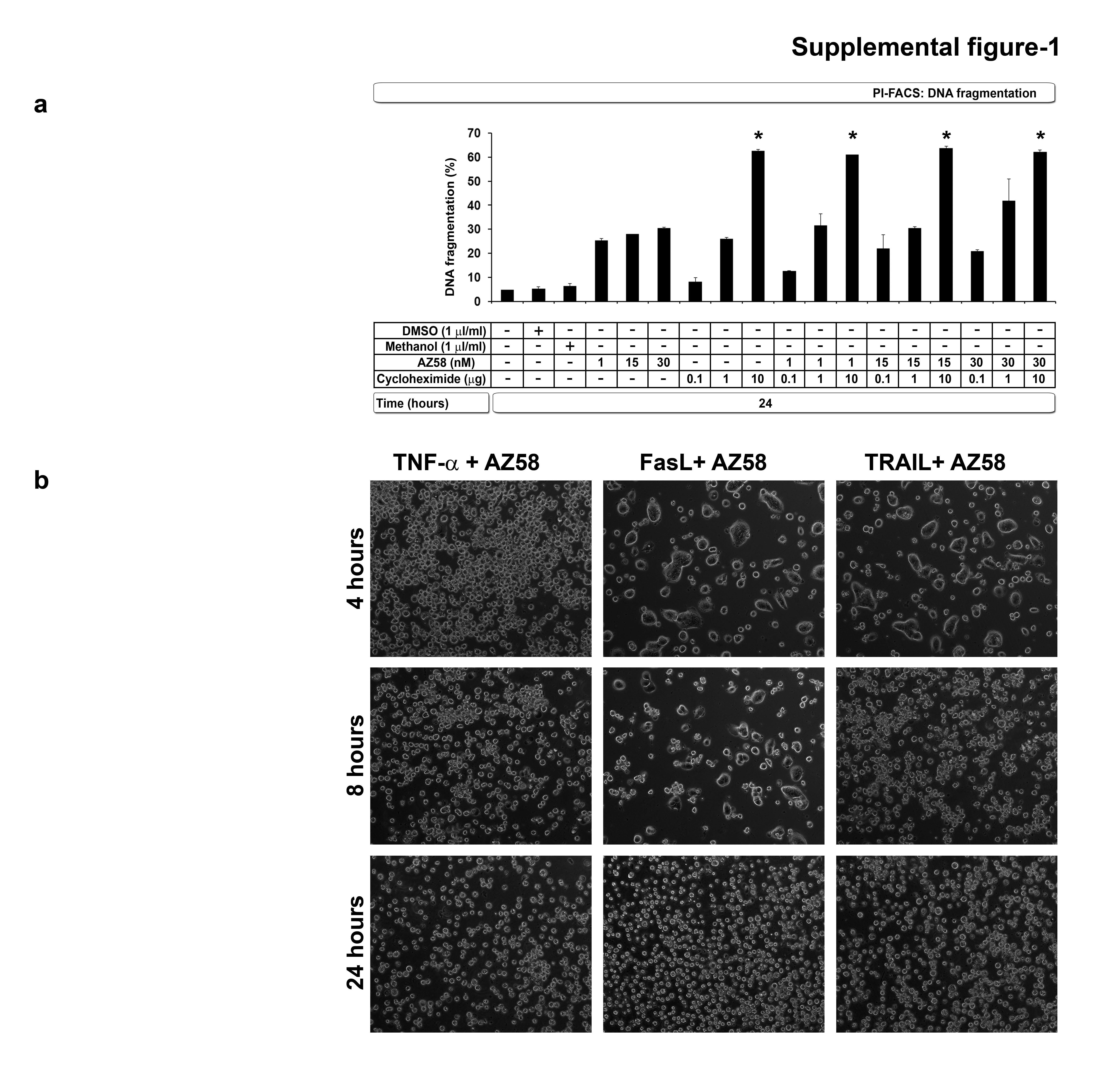
**

**Supplemental Figure 1. Cycloheximide or combinations of TNF-α, FasL, TRAIL with AZ58 induces robust apoptosis, pyknosis, blebbishield formation in RT4v6 cells**

**a.** CHX and AZ58 target the same populations of cells as no additive or synergistic effect is observed in DNA fragmentation upon incubation with CHX and AZ58 combinations (n=3). *Not significant. **b.** TNF-α and TRAIL in combination with AZ58 induce earlier pyknosis than FasL in combinations with AZ58 although all cells undergo pyknosis at 24 hr.

**
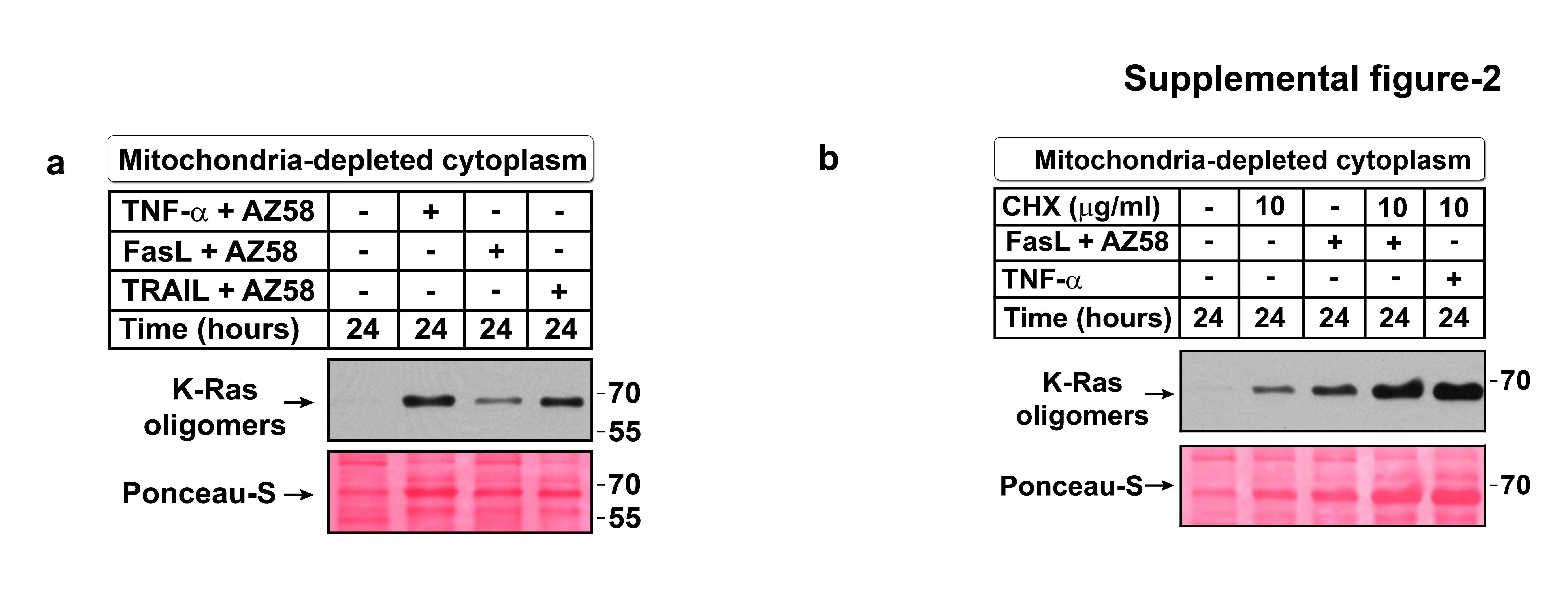
**

**Supplemental Figure 2. Detection of MOMP associated K-Ras oligomers in mitochondria depleted cytoplasm of RT4v6 cells treated with TNF-α, FasL, and TRAIL in combination with AZ58 or CHX**

**a.** Detection of K-Ras oligomers in mitochondria-depleted cytoplasm of RT4v6 cells. **b.** CHX augments cytoplasmic K-Ras oligomers as detected in mitochondria-depleted RT4v6 cytoplasm.

**
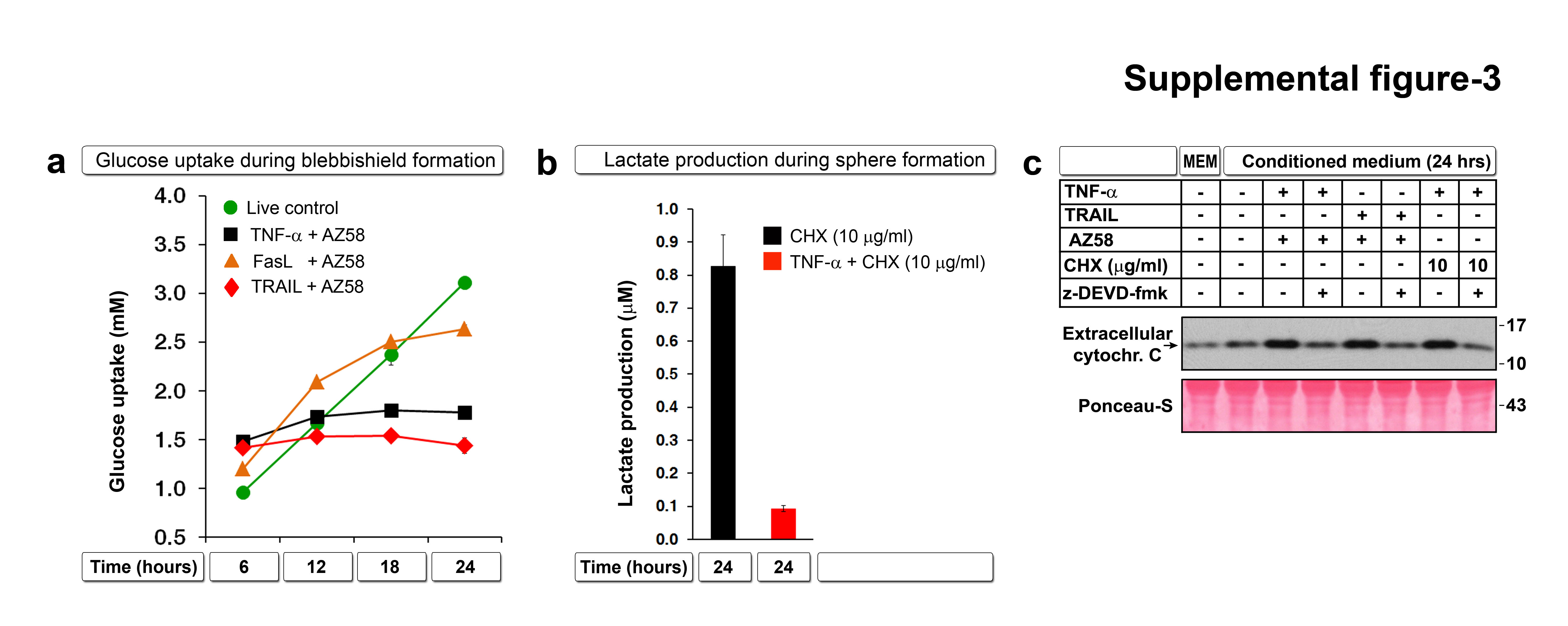
**

**Supplemental Figure 3. Glucose uptake, lactate production, and secondary necrosis detection in RT4v6 cells during blebbishield formation and sphere formation**

**a.** Glucose uptake during blebbishield formation as assessed by glucose levels in conditioned media. FasL+AZ58 had levels of glucose uptake similar to those of live control cells (n=3). **b.** Lactate production analysis from the conditioned media collected from sphere formation experiment incubated with blebbishields generated from various treatment conditions as indicated. TNF-α+CHX drastically downregulates lactate production (inhibition of glycolysis) (n=3). **c.** Detection of secondary necrosis in RT4v6 cells treated as indicated, by collecting conditioned media and comparing it with control medium to assess the release of cytochrome-C into the extracellular space (medium). Control medium, which was not incubated with cells, also has cytochrome-C (possible source is the RBC/leukocyte lysis during FBS production), and secondary necrotic conditions increase the level of cytochrome-C whereas z-DEVD-fmk reduces the cytochrome-C back to the level of controls.

**
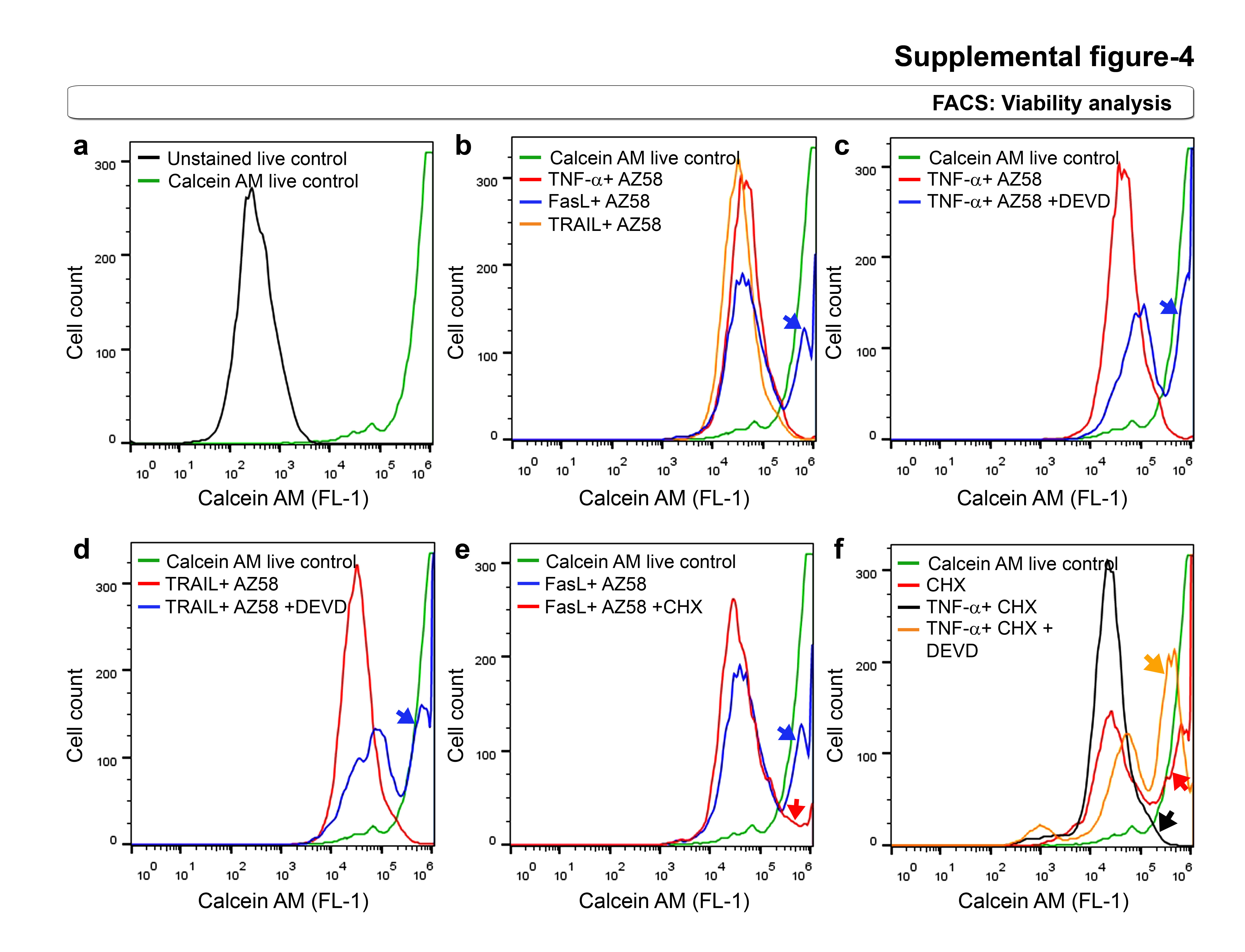
**

**Supplemental Figure 4. Viability analysis of blebbishields generated under various treatment conditions (caspase inhibition rescues viability whereas CHX abrogates it)**

This figure is associated with main Figure 5K, showing results of experiments done in RT4v6 blebbishields without trypsinization (except live controls and unstained controls) and represented as panels A-F to reduce complexity (n=3). **a.** Calcein AM staining indicates viable cells which are differentiated from auto-fluorescence of unstained cells. **b.** TNF-α+AZ58 and TRAIL+AZ58 abrogate viability (compared to control), whereas part of FasL+AZ58 blebbishield population is viable (blue arrow). **c.** Caspase-3 inhibitor rescues the viability of TNF-α+AZ58-generated blebbishields (blue arrow). **d.** Caspase-3 inhibitor rescues the viability of TRAIL+AZ58-generated blebbishields (blue arrow). **e.** CHX targets the viable blebbishields generated by FasL+AZ58 (blue vs. red arrows). **f.** CHX-generated blebbishields are partially viable (red arrow) and are killed by inclusion of TNF-α with CHX (black arrow). Caspase-3 inhibitor rescues viability of blebbishields generated by TNF-α+CHX (yellow arrow). CHX = 10μg/ml.

**Supplemental movie legends**

**Supplemental Movie S1. Caspase-3 inhibition blocks secondary necrosis but does not prevent pyknosis and blebbishield formation**

RT4v6 cells were plated in 6 well plates for 24 hr and treated with TNF-α plus Smac mimetic AZ58 with or without z-DEVD-fmk and imaged up to 8 hr in the presence of 37^o^C, 5% CO2 and humidity. Please note that z-DEVD-fmk does not prevent pyknosis but blocks secondary necrosis. Both time-lapse videos were shot at same conditions with identical frame rate and duration.

**Supplemental Movie S2. TNF-α plus CHX combination induces rapid apoptosis and secondary necrosis than CHX alone**

RT4v6 cells were plated in 6 well plates for 24 hr and treated with 10 μg/ml CHX with or without TNF-α and imaged in the presence of 37^o^C, 5% CO2 and humidity. Please note that the combination induced rapid apoptosis and secondary necrosis than CHX alone. Both time-lapse videos were shot at same conditions with identical frame rate and duration.

**Supplemental table S1**

Mechanisms of K-Ras induced cellular transformation (sphere formation) relevant to this study

| **Gene /protein** | **Associated genes/proteins common to this study** | **Mechanism of transformation** | **Supp. references** |
| --- | --- | --- | --- |
| **K-Ras^G12V^** | K-Ras | Mitochondrial dysfunction, metabolic switch to glycolysis |  |
| **K-Ras** | BAD, p27 | By regulating Pim-1 kinase |  |
| **K-Ras** | K-Ras, N-Myc | By transcriptional regulation |  |
| **K-Ras** | VEGFR2, Raf-1 | VEGFR2 inhibitor sorafenib (BAY-43-9006) blocks K-Ras-induced transformation |  |

**Supplemental references**

1. Hu Y, Lu W, Chen G, Wang P, Chen Z, Zhou Y, et al. K-ras(G12V) transformation leads to mitochondrial dysfunction and a metabolic switch from oxidative phosphorylation to glycolysis. Cell Res. 2012;22:399-412.

2. Xu D, Allsop SA, Witherspoon SM, Snider JL, Yeh JJ, Fiordalisi JJ, et al. The oncogenic kinase Pim-1 is modulated by K-Ras signaling and mediates transformed growth and radioresistance in human pancreatic ductal adenocarcinoma cells. Carcinogenesis. 2011;32:488-95.

3. Tchernitsa OI, Sers C, Zuber J, Hinzmann B, Grips M, Schramme A, et al. Transcriptional basis of KRAS oncogene-mediated cellular transformation in ovarian epithelial cells. Oncogene. 2004;23:4536-55.

4. Campbell PM, Groehler AL, Lee KM, Ouellette MM, Khazak V, Der CJ. K-Ras promotes growth transformation and invasion of immortalized human pancreatic cells by Raf and phosphatidylinositol 3-kinase signaling. Cancer Res. 2007;67:2098-106.
